# Supplementary material for: Keeping weight off: Mindfulness-Based Stress Reduction alters amygdala functional connectivity during weight loss maintenance in a randomized control trial
Source: PLoS One. 2021 Jan 11;16(1):e0244847. doi: 10.1371/journal.pone.0244847 (PMC7799782; doi:10.1371/journal.pone.0244847)
Supplement: S1 Fig — (DOC) [file pone.0244847.s002.doc]

**
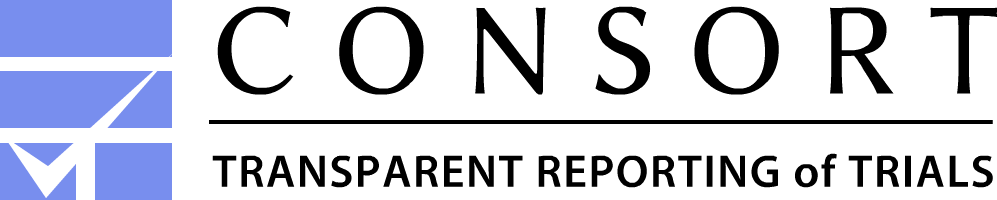
**

**CONSORT 2010 Flow Diagram**

**Allocation**

**Analysis**

**Follow-Up**

**Enrollment**

Assessed for eligibility (n= 472 )

Excluded (n= 415 )

♦  Not meeting inclusion criteria (n= 164 )

♦  Declined to participate (n= 219 )

♦  Other reasons (n= 32 )

Analysed (n= 23 )
♦ Excluded from analysis (give reasons) (n= 0 )

Lost to follow-up (give reasons) (n= 23 )

Discontinued intervention (give reasons) (n= 0 )

Allocated to intervention: HLC (n= 28 )

♦ Received allocated intervention (n= 23 )

♦ Did not receive allocated intervention (give reasons) (n= 5) Unable to complete MRI or dropped out during intervention.

Lost to follow-up (give reasons) (n= 29 )

Discontinued intervention (give reasons) (n= 0 )

Allocated to intervention MSBR (n= 29 )

♦ Received allocated intervention (n= 29 )

♦ Did not receive allocated intervention (give reasons) (n= 0 )

Analysed (n= 29 )
♦ Excluded from analysis (give reasons) (n= 0 )

Randomized (n=57 )
