# Supplementary material for: Keeping weight off: Mindfulness-Based Stress Reduction alters amygdala functional connectivity during weight loss maintenance in a randomized control trial
Source: PLoS One. 2021 Jan 11;16(1):e0244847. doi: 10.1371/journal.pone.0244847 (PMC7799782; doi:10.1371/journal.pone.0244847)
Supplement: S1 File — (DOCX) [file pone.0244847.s003.docx]

1. Title

Keeping weight off: Brain Changes Associated with Healthy Behaviors

1. IRB Review History*

NA

1. Objectives*

Mindfulness-Based Stress Reduction (MBSR) was designed to foster lifelong self-monitoring and self-regulation through increased awareness, healthy behaviors, and stress management [1-3]. Our team, which includes the developers of MBSR, is planning a clinical trial to test its effectiveness for maintenance of health behavior change. Although evidence exists for effective interventions to initiate health behavior change, evidence for their effectiveness for maintenance of behavior change is disappointing. [4-7] Based on evidence that stress and negative emotions predict worse outcomes for maintenance of health behavior change and relapse to unhealthy behaviors [8-10], and strong evidence for MBSR-based maintenance interventions for depression and addiction[11-14], we believe that MBSR may be effective for maintenance of health behavior change.

Based on our preliminary data and previous research [15], we hypothesize that MBSR’s impact on reactivity to stress will be manifested in enhanced functional integrity of the cortico-limbic network involved in modulating reactivity to stress and negative emotions. We have studied these changes using Functional Connectivity (FC) MRI**,** which quantitatively measures the strength and integrity of functional networks using fMRI. FC is responsive to changing levels of stress[16], intense training on a task[17], and meditation practice including MBSR [18, 19](and see below). Our preliminary results show that FC in the amygdala-orbitofrontal network involved in modulation of emotional/stress reactivity is strongly correlated with trait mindfulness and is enhanced by MBSR.

To characterize the FC changes in response to MBSR and the comparison condition, we will randomize a sample of 100 participants who have intentionally lost > 5% of their body weight during the previous year to MBSR or an attention control specifically designed to be structurally equivalent to MBSR. R34 participants will be randomized into the two conditions (MBSR vs. attention control).. FC, psychological factors, health behaviors, and BMI (body mass index) will be measured at baseline and 8 weeks. Psychological factors, health behaviors, and BMI will also be measured at 6 months.

To address the possibility that an imbalanced distribution of baseline FC could mask important findings, we will employ a non-stratified permuted-block randomized design with an interim analysis of the distribution of baseline functional connectivity. The permuted-block design will take a block size of 10 with an allocation ratio of 1:1 to assure equal assignment of participants to each study condition. The interim analysis will occur after 40 participants have been enrolled. At this point we will examine the mean, median, and full distributions of functional status for both study groups. If important imbalances are found, we will consider a range of responses including selective enrollment of participants who have baseline functional connectivity with a specified range, covariate-adaptive randomization techniques, and post-hoc statistical adjustment. (We do not plan to examine outcomes at this interim analysis, so adjustment for “multiple looks” will not be necessary.) Results of the interim analysis will be shared with the extramural reserach team at NCAM for guidance about the best approach.

**Aim 1. Characterize functional connectivity changes in response to MBSR and the comparison condition.**

Hypothesis 1: Participants randomized to the MBSR condition will experience greater increases in functional connectivity from baseline to 8 weeks post-intervention.

Hypothesis 2: Participants with higher baseline functional connectivity will show less change in response to MBSR.

**Aim 2. Investigate the association of functional connectivity change with changes in psychological factors and maintenance of weight loss at 8 weeks and 6 month follow-up.**

Hypothesis 3: Change in functional connectivity will be correlated with changes in depressive symptoms.

Hypothesis 4: Change in functional connectivity will be inversely correlated with changes in weight (BMI).

As secondary aims, we will use mediation analysis to determine how change in FC is explained by (1) class attendance, (2) self-reported time in homework practice, (3) self-reported time for each specific component of the multifaceted training program, and (4) trait mindfulness. An exploratory aim is to examine correlations of change in FC with changes in additional psychological factors (perceived stress, trait anger, trait anxiety, positive affect) and health behaviors (healthy eating, physical activity, sleep quality).

**Aim 3. Assess changes in BMI at 6 & 12 months to obtain preliminary measures of effect size and variability by study group to inform sample-size calculations for the randomized trial.**

1. Background*

Unhealthy behaviors such as overeating and sedentary lifestyles are major contributors to cardiovascular disease, cancer, type 2 diabetes and other chronic conditions. They have produced a rapid rise in obesity that threatens to reverse recent gains in life expectancy and account for a large percentage of premature deaths in the U.S. Although there is evidence for the effectiveness of interventions for initiating health behavior change, these interventions have shown only limited ability to affect significant, long-term behavioral changes in the majority of adults. [4-7] In part this may be attributable to failure to adequately address the effects of perceived stress and emotional reactivity on relapse to unhealthy behaviors and failure to maintain long-term behavior change. Perceived stress and symptoms of emotional reactivity (depression, anxiety, anger) are linked to unhealthy lifestyle behaviors [20-23] and predict worse outcomes in maintenance studies (see for reviews [8-10]). Indeed, studies of health behavior change have demonstrated that perceived stress[24-26] and indices of emotional reactivity such as anxiety [27-29], depression, and anger [30-32] are associated with poor outcomes.

Research supports the efficacy of MBSR for reducing stress and emotional reactivity – including perceived stress (see [33, 34]for reviews), anxiety [35-38] [39], depressive symptoms [36, 38-41], and anger[42, 43] (& see below) – and for increasing positive affect [37, 44], which is associated with enhanced emotional regulation [45-47]. In addition, rigorous clinical trials of an adaptation of MBSR – Mindfulness-Based Cognitive Therapy (MBCT) – have shown it to be as effective as maintenance antidepressants for prevention of depression relapse[12, 14], and preliminary studies support the efficacy of Mindfulness-Based Relapse Prevention (MBRP) for prevention of relapse to substance abuse[11]. Therefore, we hypothesize that MBSR may be effective for maintenance of health behavior change through promotion of skills for reducing emotional and behavioral reactivity to stressors which will reduce the risk of relapse to unhealthy behaviors. We believe that following successful initiation of health behavior change, MBSR may improve the ability to maintain these changes. We will test this hypothesis in a pilot randomized controlled trial of MBSR in people who have completed a weight loss program.

The current study will characterize the FC response to elucidate the specific effects of MBSR on emotional and stress reactivity. We hypothesize that this response will predict sustained changes in health behaviors at 6-month follow-up, thereby reducing reliance on self-reported measures and providing an objective measure for identifying patients most likely to benefit and those who may need a modified intervention. We will employ a rigorous methodology to demonstrate that: (1) functional connectivity is sensitive and specific to MBSR training and (2) post-MBSR changes in functional connectivity of the amygdala-orbitofrontal circuit is correlated with meaningful changes in emotional reactivity and long term (six months) health behaviors and maintenance of weight loss. In addition, we will conduct exploratory analyses to characterize the effect of time spent in practice and specific intervention components on changes in FC.

Preliminary Data: We recruited 14 participants from the community and 6 participants from MBSR classes at the UMass Center for Mindfulness (mean age 31 yrs, SD 14.6). All 20 participants received resting-state fMRI scans and psychometric measures of mindfulness (Kentucky Inventory of Mindfulness Scale, KIMS), Trait Anger (STAXI-II), and Perceived Stress (PSS). The 6 MBSR participants completed baseline measures <1 week pre-MBSR and again <1 week after exposure to MBSR.

Participants were scanned on the 3.0Tesla Phillips scanner in the UMMS Advanced MRI Center using standard resting state parameters. We used amygdala as the seed region for examining FC for all participants. Strong connections were seen between amygdala and orbitofrontal cortex (OFC). The spatial pattern of functional connectivity was consistent with structural and functional studies. As expected, amygdala functional connectivity covaried with Trait Mindfulness (KIMS scores). and Trait Anger showed an inverse association with amygdala-orbitofrontal FC.

The MBSR participants showed meaningful changes in pre-post emotional reactivity and mindfulness measures in the expected direction: Trait Anger (STAXI-II) decreased 27% and Perceived Stress (PSS) 11%, while Positive Affect increased 19% and trait mindfulness (total KIMS) increased 27%. In addition, amygdala-orbitofrontal FC increased after exposure to MBSR. Using voxel-by-voxel comparison with two-sample t-test, functional connectivity between amygdala and middle OFC was significantly strengthened after MBSR training (p<.005).

Taken together, both our psychometric data and FC analysis in amygdala-orbitofrontal circuitry suggest that MBSR training produces greater top-down control of stress and emotional reactivity. In agreement with the pre-post MBSR analysis, our baseline correlation analysis, demonstrates a negative association of emotional reactivity (trait anger), and a positive association of trait mindfulness with amygdala-orbitofrontal FC.

These data are consistent with the proposed hypothesis that a critical component of MBSR is the enhancement of emotional regulation and with our proposed model for the utility of a neuroimaging biomarker for MBSR. Specifically, current data support the hypothesis that emotional regulation is a critical component of MBSR that can serve as a valuable biomarker and predictor of long-term maintenance of healthy behaviors.

Although other studies have examined resting state functional connectivity in relation to mindfulness, [18, 19] this study is highly innovative because our approach is based on a specific hypothesis about specific resting state changes in the cortico-limbic network involved in modulation of the stress response and the critical role this network plays in health behaviors. This study will help to elucidate the mechanism of MBSR by identifying stable changes in brain circuitry that correspond with lasting effects of MBSR, and provide critical information on effect size for a large clinical trial of the effectiveness of MBSR for maintenance of weight loss.

1. Inclusion and Exclusion Criteria*

Inclusion criteria: males and females age 25-55 years, right- or left-handed; under the care of a primary care physician for at least the last year prior to screening; ability to communicate with the research staff by telephone; intentional loss of > 5% of body weight in year prior to study entry, satisfied with weight loss and intending to maintain the weight loss; BMI > 25 kg/m2 in the past 2 years and greater than 20.5 kg/m2 at time of study entry. To verify self-reported weight loss, participants will be required to have their healthcare provider, personal trainer or weight-loss counselor complete and sign a form indicating the amount and timing of their weight loss, or if not available, a dated photograph or weight loss diary.^[48]^

Exclusion criteria: weight > 300 lbs. (capacity of MRI scanner); prior participation in an MBSR course; regular meditation practice (or any other form of meditative practice, such as yoga, Tai Chi or contemplative prayer) for more than an average of 20 minutes a week within the past 2 years; participants with a serious psychiatric, cognitive or medical disorder which could interfere with completion of the study, or a history of alcohol (>14/week or >4 drinks at any one time for a male, or >7 drinks/week or >3 drinks at any one time for a female) or substance abuse (high frequency and problems caused) or dependence in the past 6 months; any conditions which are incompatible with MRI and structural brain damage as determined by an independent neuroradiologist based on T1W 3D TFE sagittal and T1W FFE axial images; history of an eating disorder; diabetes mellitus or medications for diabetes mellitus; any medication that affects weight (e.g. weight loss medications, corticosteroids, antipsychotics); history of weight loss surgery; participation in another weight management research study; regain of >10% of the weight lost during the previous year in the 2 months prior to study entry; intentionally trying to lose additional weight at study entry. For their own safety and comfort, subjects must not have claustrophobia, or any MRI incompatible implants. Specifically, people with the following will not be eligible to participate:

• Cardiac pacemakers, defibrillators

• Aneurysm clips and other vascular stents, filters, clips or other devices

• Prosthetic heart valves

• Other prostheses

• Neuro-stimulator devices

• Implanted infusion pumps

• Cochlear (ear) implants

• Ocular (eye) implants or metal fragments in eyes

• Exposure to shrapnel or metal filings (sheet metal workers, welders, and others)

• Other metallic surgical hardware in vital areas

• Certain tattoos with metallic pigments

We will not include any of the following special populations:

- Adults unable to consent
- Individuals who are not yet adults (infants, children, teenagers)
- Pregnant women
- Prisoners

1. Study-Wide Number of Subjects*

UMMS is the only study site. The maximum number of subjects to be recruited is 100.

1. Study-Wide Recruitment Methods*

NA (see **24** below)

1. Study Timelines*

The duration of each subject’s participation in the study will be 15 months.

The duration anticipated to enroll all study subjects is 14 months.

The estimated date to complete this study is Dec 31, 2016.

Timeline:

| **Year 1 Tasks Months:** | ***1*** | ***2*** | ***3*** | ***4*** | ***5*** | ***6*** | ***7*** | ***8*** | ***9*** | ***10*** | ***11*** | ***12*** |
| --- | --- | --- | --- | --- | --- | --- | --- | --- | --- | --- | --- | --- |
| Hire RA, develop databases, train RA in HLC |  |  |  |  |  |  |  |  |  |  |  |  |
| Recruit 1^st^ cohort (N=25), screen, randomize, baseline data collection |  |  |  |  |  |  |  |  |  |  |  |  |
| 1^st^ cohort classes; 8-week data collection/entry |  |  |  |  |  |  |  |  |  |  |  |  |
| Recruit 2^nd^ cohort (N=25), screen, randomize, baseline data collection |  |  |  |  |  |  |  |  |  |  |  |  |
| 2^nd^ cohort classes |  |  |  |  |  |  |  |  |  |  |  |  |
| **Year 2 Tasks Months:** | ***13*** | ***14*** | ***15*** | ***16*** | ***17*** | ***18*** | ***19*** | ***20*** | ***21*** | ***22*** | ***23*** | ***24*** |
| 2^nd^ cohort 8-week data collection/entry |  |  |  |  |  |  |  |  |  |  |  |  |
| Recruit 3^rd^ cohort (N=25), screen, randomize, baseline data collection |  |  |  |  |  |  |  |  |  |  |  |  |
| 3^rd^ cohort classes; 8-week data collection/entry |  |  |  |  |  |  |  |  |  |  |  |  |
| 1^st^ cohort 6 mo. data collection/entry |  |  |  |  |  |  |  |  |  |  |  |  |
| 2^nd^ cohort 6 mo. data collection/entry |  |  |  |  |  |  |  |  |  |  |  |  |
| Recruit 4^th^ cohort (N=25), screen, randomize, baseline data collection |  |  |  |  |  |  |  |  |  |  |  |  |
| 4^th^ cohort classes; 8-week data collection/entry |  |  |  |  |  |  |  |  |  |  |  |  |
| 1^st^ cohort 12-m0. data collection (phone) |  |  |  |  |  |  |  |  |  |  |  |  |
| 3^rd^ cohort 6 mo. data collection/entry |  |  |  |  |  |  |  |  |  |  |  |  |
| **Year 3 Tasks Months:** | ***25*** | ***26*** | ***27*** | ***28*** | ***29*** | ***30*** | ***31*** | ***32*** | ***33*** | ***34*** | ***35*** | ***36*** |
| 2^nd^ cohort 12-m0. data collection (phone) |  |  |  |  |  |  |  |  |  |  |  |  |
| 4^th^ cohort 6-month data collection/entry |  |  |  |  |  |  |  |  |  |  |  |  |
| 3^rd^ cohort 12-m0. data collection (phone) |  |  |  |  |  |  |  |  |  |  |  |  |
| 4^th^ cohort 12-mo. data collection (phone) |  |  |  |  |  |  |  |  |  |  |  |  |
| Data analysis |  |  |  |  |  |  |  |  |  |  |  |  |

1. Study Endpoints*

This is a pilot study designed to collect data about effect size and variability by study group to inform sample-size calculations for the randomized trial. Therefore the primary and secondary endpoints are exploratory. We will examine between-individual means and variance for weight and body mass index at each point in time and within-person, over-time weight trajectories to determine variance and correlations for the entire cohort and separately by study group. We will compare over-time changes in weight and body mass index by study group, and explore potential moderating factors such as patient baseline variables including functional connectivity.

As there are no known risks associated with the interventions being investigated there is no Data Safety Monitoring Plan and no safety endpoints for this study.

1. Procedures Involved*

At the first research visit (T0 in Table 1 below; 1.5-2 hours), participants will meet with a psychiatrist who will complete the medical history and SCID-DSMIV, and the Research Assistant who will collect demographic data and height and weight and administer questionnaires, and have the first MRI. Participants will then be randomly assigned to either the MBSR or HLC condition which will begin within 4 weeks. After completing the interventions, participants will return for the second research visit (T1 in Table 1 below; 1.5-2 hours) to repeat the MRI, questionnaires and weight measurement. Participants will be scheduled for the third research visit 6 months after completing the intervention (T2; 0.5-1 hour) to complete the questionnaires, including continued use of the mindfulness practices (see Practice Log 6&12 mo). and weight measurement. One year after completing the intervention (T3; 5 minutes), participants who agree to be contacted by phone will be asked to self-report their weight and continued use of the mindfulness practices.

Experimental Condition: Mindfulness-Based Stress Reduction (MBSR)

MBSR is a psycho-educational program that consists of training in formal and informal mindfulness practices, information about the role of good nutrition, rest and exercise in health, and information about the role played by thoughts and emotions in health. It was developed at UMass by Dr. Jon Kabat-Zinn and has been disseminated worldwide. MBSR is taught in eight 2 ½ hour classes and one all-day retreat led by a teacher in a classroom format. The curriculum includes classroom activities and homework assignments including the expectation to engage in the formal meditation practices for 45 minutes daily six days per week and in informal practices during daily life. Two compact discs containing four 45 minute instructions for home practice are provided. Classroom activities teach participants to focus attention through formal meditation (sitting meditation, body scan, mindful yoga, walking meditation) and informal meditation techniques such as bringing awareness of the present moment to various aspects of daily life such as eating, walking and listening to others. Participants are taught to be aware of their immediate thoughts, emotions and sensations and to let them come and go in awareness without attempting to change, suppress or elaborate on them. Participants learn to apply this practice to daily events including stressful experiences in order to avoid reflexive or conditioned reactions that can be emotionally arousing or unhelpful.

For the MBSR classes participants will be enrolled in on-going MBSR classes provided by the Center for Mindfulnestaught by certified teachers from the UMass Center for Mindfulness . Certified teachers have completed a rigorous training and certification and their teaching observed by senior teachers to ensure fidelityTo further assure fidelity each class will be reviewed in weekly sessions with Co-I Dr. Santorelli. In addition, the PI’s will convene the entire clinical research team routinely to monitor the overall fidelity of the MBSR intervention for this study.

Attention Control: Healthy Living Course

The control condition will consist of the implementation of the Healthy Living Course (HLC). The HLC was developed by our outside consultant (Sarah Reiff-Hekking, Ph.D.) who is not involved in the design, conduct or reporting of this research. Dr. Reiff-Hekking’s involvement will be solely limited to training the RA in how to deliver the course to subjects. Direct supervision of the RA may be provided by Co-I Rosal or her delegate. HLC consists of 12 weekly classes of 2 hours. The first 8 sessions consist of lectures and discussion on the following topics: healthy living, healthy eating, physical activity and health, sleep and health, stress management, time management, and unhealthy behaviors (smoking, drinking). The HLC will serve to control for attention and other nonspecific factors including staff interactions, psychoeducation about health and stress management, classroom format, homework, group process, and data collection.

*Outcomes*

Seed-based resting-state functional connectivity MRI

All MRI’s will be acquired on the 3T scanner (Philips Achieva) in the UMMS Advanced MRI Center.  3D high-resolution structural T1-weighted MR images will be obtained to provide anatomical landmarks. Following the structural imaging, resting-state fMRI data will be collected. Participants will be instructed to remain relaxed with eye closed as fMRI images are continuously collected for 10mins. The duration of the entire MRI procedure will be 30 min.

Self-reported measures

**Health behaviors**: We will use the Eating Behavior Inventory [49] to measure eating behaviors and the Paffenbarger Physical Activity Scale [50] to measure Physical Activity The Pittsburgh Sleep Quality Index (PSQI) will be used to assess overall sleep quality and sleep habits in the past month.[51]


**Markers of stress/emotional reactivity and positive affect**: Psychological outcomes shown to significantly impact health behavior outcomes will be measured. [9, 52, 53] We will assess indices of stress (perceived stress) and emotional reactivity (depression, anxiety, anger), positive affect, and disinhibition of eating using the following measures: the Perceived Stress Scale (PSS-14) [54], the Center for Epidemiologic Studies Depression Scale (CES-D)[55], the State-Trait Anxiety Inventory – trait version (STAI-T) [56], the State-Trait Anger Expression Inventory (STAXI-II) trait anger subscale[57], the the Satisfaction with Life Scale [58, 59] the Emotion Regulation Questionnaire [60], and the Internal Disinhibition Subscale[61] .

*Potential mediators and confounds*

- *Intervention engagement:* Class attendance will be recorded by instructors and tracked in the study. At the baseline visit participants will be given a log to record frequency of homework practices, including formal practices (body scan, yoga and meditation) and informal practices (awareness of the breath in daily life), and given instructions regarding adherence and maintaining practice logs. Logs will be collected at the post-intervention visit and will indicate number of minutes and days engaged in formal and informal practice each week. Prior to the 6 and 12 month follow-up participants will be sent a practice log for recording the approximately frequency and duration of practice and comments about what benefit if any they have continued to derive from the training.
- *Trait mindfulness:* Although the utility of self-reported measures of mindfulness is controversial [62], we will use the Five-Facet Mindfulness Questionnaires (FFMQ) [63] to assess the association of change in our biomarker with trait mindfulness..
- *Credibility and Expectancy:* Immediately after the initial session for each intervention participants will complete the Credibility/Expectancy Questionnaire CEQ [64] modified slightly to substitute the word “Class” for “Therapy” in the instructions. Questions were rated on a 1-to 9-point Likert scale. This scale has been widely used in behavioral therapy studies and shown to have good psychometric properties.[64]

*Assessment and intervention schedule*

Participants randomized to MBSR will be exposed to the 8 week MBSR course. Outcome measurement will be parallel for the two groups at 8 weeks, including FC by fMRI, BMI, health behaviors and psychological assessments. Questionnaires and MRI scans will be obtained on the same day. BMI, health behaviors and psychological measures will also be collected at 6 months follow-up. Self-reported weight will be collected by phone at 12 months follow-up.

**TABLE 1. Assessment schedule:T0=baseline; T1=8 weeks; T2=12 weeks; T3=6 mos.**

| **Measure** | **T0** | **T1** | **T2** | **T3** |
| --- | --- | --- | --- | --- |
| Selection Factors (N=100) Demographics; SCID-DSMIV*;* inclusion/exclusion criteria | **X** |  |  |  |
| Credibility and Expectancy: CEQ | **X** |  |  |  |
| fMRI scans | **X** | **X** |  |  |
| BMI, Health behaviors & Psychological measures | **X** | **X** | **X** |  |
| Mediators: Attendance, practice logs |  | **X** |  |  |
| BMI self-report by phone |  |  |  | **X** |

*Drop-outs*

The PI or Research Assistant will make up to three attempts to contact participants if they appear to drop-out by not appearing either for research visits or for intervention classes. We will ask participants if they intend to drop out of the study and if so their reasons for dropping out. No attempt will be made to persuade or induce them to continue.

Procedures to minimize risk of harm: Subjects will be asked whether they have devices that can be affected by MRI, and if so, they will not be able to participate in this study. Significant risks also can arise if ferromagnetic materials (this includes many types of common metal objects) are brought into the high magnetic field environment of the scanner and immediate vicinity, as they can become hazardous projectiles. These types of items are not permitted in the scanning area. All staff of the Advanced MRI Center are trained to work in the MR environment and protect the safety of subjects. The MR exams are painless, and except for the pulsating sounds, subjects will not be aware that MR scanning is taking place.

This study will be conducted in a 3T MR scanner which has been approved for research and clinical studies in children and adults by the FDA. The technologist will be able to hear subjects at all times and subjects are free to end the procedure at any time. In rare cases, a very slight, uncomfortable tingling of the back due to the rapid switching of the magnetic field has been reported during certain types of scans. In case subjects have such a sensation, they are asked to report this immediately, so the scan can be changed to avoid this. The sounds that subjects hear inside the scanner are the normal operating sounds the scanner makes while it takes pictures and collects data. While the sounds may be annoying, the intensity is not harmful to their hearing. However, subjects will be given a pair of earplugs and headphones to wear which will muffle the sounds and allow communication with the scanner operator.

1. Data and Specimen Banking*

NA

1. Data Management*

Data Management: We will enter the data using the REDCap™ system, a nationally used secure, web-based database application (http://project-redcap.org) that provides: 1) an intuitive interface, 2) audit trails for tracking data manipulation and export, 3) procedures for importing data from external sources, 4) easy development of web survey tools and 5) automated export procedures to various statistical packages. We will program the system with validation rules at time of entry and comprehensive edits conducted after data have been submitted to the main data base. Edits will be programmed into the initial data entry screens to check for validity, consistency, and normal range values. Edit queries will be generated and resolved by the Masters-level R.A. (TBH) with corrections posted to database through REDCap, which enforces an audit trail for all changes. A comprehensive data dictionary will be created which will specify each data element, variable type, units, range, and missing data code(s). Training and data collection schedules and reports will be maintained and discussed in team meetings. REDCap system uses MS SQL Server as the underlying database system at UMass. Data stored on secure server in UMass HIPAA-compliant data center with daily back-up. For analysis, data exported from REDCap system as SAS data sets and merged within SAS to create official study analysis. All reports and analyses generated using SAS. Data files (and SAS programs) used for reports, presentations, or publications are archived as required. REDCap will also be used for patient tracking and protocol adherence so that missing data can be identified and captured promptly. Reports of missed visits, missed data collection, etc., will be generated directly from REDCap and used by research staff to resolve missing data problems. Co-I Dr. Allison (M.D., M.Sc. Epi) or his delegate will supervise data management. Quality assurance/control measures will be instituted. For data entry/ management, periodic comparison of data samples from the database to the source documents will be conducted and, if there is > 1% discrepancy, further comparisons will be conducted to determine if random or systematic. For MRI measures, data will be analyzed blind to intervention group by the two fMRI experts (Co-I’s King and Zhang) who will perform cross-validity checks. We will develop a program to re-measure a small subset of questionnaires for QA purposes and to identify any systematic deviations from protocol by research staff.

Data Analysis:

Overview. Statistical analyses will begin by examining univariate statistics and distributions. Graphic and panel techniques suitable for longitudinal data will examine over-time changes. We will examine the balance of participant characteristics by study groups and account for observed imbalances with multivariable adjustment.

Hypothesis 1: The main effect will be differential over-time change in FC for the intervention versus comparison group. Mixed models will represent the clustering of observations within participants as a random intercept. Multivariable models will be estimated with the Stata generalized linear latent and mixed model (GLLAMM) module,(15-16) taking a Gaussian family and an identity link. Because of the small number of clusters and potentially unbalanced data from uneven loss to follow up across groups and time, parameter estimation will use adaptive quadrature.

The model for Hypothesis 1 will be parameterized with indicator variables for study group, time, and a group-time interaction. The group-time interaction coefficient will represent differential over-time change in the outcome (FC) for the intervention versus comparison group, with positive values favoring the intervention. To preserve the power of randomization, hypothesis will be analyzed on an **intent-to-treat** basis.

Hypothesis 2. This hypothesis seeks to uncover an interaction between baseline FC and pre-post intervention change in FC. This interaction will be examined using three approaches. First, we will focus only on the intervention group and examine the bivariate association of over-time change in FC with baseline FC, both graphically and statistically with the Spearman correlation coefficient if linearity assumptions are reasonably met. We will also use simple ANOVA and trend tests to examine change in FC across categories of baseline FC. Finally, for this first approach we will use techniques of regression modeling described above to predict change in functional status while accounting for important clinical and demographic covariates. For the second approach we will use both study groups and repeat the analysis described above for Hypothesis 1 separately within pre-defined strata of baseline FC. Lacking a clear literature-based definition of how to interpret the FC magnitude, we will first define the strata based upon tertiles; however, we recognize that such an approach may be limited by small participant numbers. Therefore, we will explore other approaches such dichotomizing baseline FC at the median. For the third approach,we will use both study groups to expand the analysis from Hypothesis 1 to develop a more complex model that include a three-way interaction between group, time, and baseline. Marginal means and confidence intervals from the delta method (Taylor linearization) will be calculated to aid in interpretation of the resulting complex models. Interpretation of this model will be facilitated by a graphical display of continuous over-time change in functional connectivity by study group and by baseline level of FC.

Additional Analyses. Our basic approach to Hypothesis 3 and 4, the mediation analysis, and the ancillary analyses will follow the plan outlined in the latest version of the peer-reviewed proposal. We will begin by examining bivariate correlations. Next, sequential model-building strategies will adjust for sociodemographics factors, clinical characteristics, and psychological attributes. A full range of diagnostic tests will detect and allow us to respond to heteroskedasticity, non-linear response, excessive multicollinearity, and extreme influence. Given the modest sample size, we will take caution to avoid over-fitting.

Missing data. Missingness not at random may introduce important bias into both primary and secondary analyses. The most important defense against bias from missing data is advanced planning.(17-18) During the initial project phases, we will use sound principles of data collection and quality control (Section C.4 of peer-reviewed proposal) to minimize missing data. When faced with missing data at the point of analysis, several approaches from the basic to more complex will be considered. First, participants with missing data will be compared to those without. Sensitivity analyses will be used to estimate the bound of potential bias introduced by missingness. Under the missing-at-random assumption, multiple imputation(19) or non-parametric, doubly robust imputation.(20) may be used to generate plausible values of missing data while appropriately accounting for the additional uncertainty introduced by the missingness. Inverse probability weighting and pattern-mixture modeling are alternative techniques.(21-22)

Multiple Testing. Although significant controversy surrounds the issue of false positive testing,(23) we will follow leading-edge methodology being used in the field of genomics to control the false positive discovery rate (FDR).(24-28) Unlike the Bonferroni-family of corrections applied to traditional hypothesis testing, the FDR approach is designed to assure that (on average) only a certain fraction of comparisons declared to be statistically significant will be false positives. Such an approach allows control of alpha error without greatly inflating beta error. In contrast, the Bonferroni family of approaches has been criticized as causing severe losses of power,(29-31) which could have drastic ramifications for studies with limited sample sizes.

1. Provisions to Monitor the Data to Ensure the Safety of Subjects*

This research involves no more than Minimal Risk to subjects. Subjects are asked about suicidality during the screening by a psychiatrist. If responses indicated presence of suicidality the psychiatrist would make an immediate referral and/or escort the subject to the Emergency Room for evaluation. Research measures do not ask about suicidality. However, the CES-D assesses other symptoms of depression. If responses to these questions indicate presence of severe depression, the P.I., a psychiatrist, would immediately be contacted to evaluate the subject for safety and make a referral if indicated. Standard risk management procedures would be used if any clinical risk or safety information arises in the course of data collection.

1. Withdrawal of Subjects*

We do not foresee any circumstances under which subjects will be withdrawn from the research without their consent. Only participants who request withdrawal from the study will be withdrawn. Subjects who withdraw prior to one of the data collection points (T1-T3) will be contacted to inquire about their reasons for dropping out as this may provide valuable information about the study design and/or the interventions. They will also be asked if they would be willing to be contacted for subsequent outcome measures. When faced with missing data at the point of analysis, several approaches from the basic to more complex will be considered. Participants with missing data will be compared to those without. Sensitivity analyses will be used to estimate the bound of potential bias introduced by missingness.

1. Risks to Subjects*

We do not foresee any risks to subjects. Magnetic resonance (MR) technology does not use ionizing radiation like an X-ray. Instead, it uses strong magnetic fields and radio waves to collect the images and data. Subjects will be asked whether they have devices that can be affected by MRI, and if so, they will not be able to participate in this study. Significant risks also can arise if ferromagnetic materials are brought into the high magnetic field environment of the scanner and immediate vicinity, as they can become hazardous projectiles. These types of items are not permitted in the scanning area. The MR exams are painless, and except for the pulsating sounds, subjects will not be aware that MR scanning is taking place.

With proper safety precautions in terms of the avoidance of metal objects, there are no known health risks associated with MRI. The safety of MRI is reflected in the fact that it is used in standard medical practice without the requirement for informed patient consent. Most people experience no ill effects from the magnetic field, but some report claustrophobia, dizziness, mild nausea, headaches, a metallic taste in their mouth, double vision, or a sensation of flashing lights. These symptoms are transient and resolve quickly after the subject exits the scanner. The technologist will be able to hear subjects at all times and subjects are free to end the procedure at any time. In rare cases, a very slight, uncomfortable tingling of the back due to the rapid switching of the magnetic field has been reported during certain types of scans. Subjects are asked to report this immediately so the scan can be changed to avoid this. These symptoms, if present, disappear shortly after leaving the MR machine. Subjects may feel cramped inside the scanner. The scanner is noisy, but does not harm hearing. For comfort, subjects will be given earplugs to muffle the noise.

Psychiatric and psychological test data will be collected on all participants. There is a slight chance that some participants could experience discomfort while answering the questionnaires. As these will be obtained by highly experienced mental health providers, standard risk management procedures would be used if any clinical risk or safety information arises in the course of data collection. The likelihood of such discomfort is minimal based on our previous experience administering these questionnaires. If they do occur the duration is likely to be quite brief, on the order of minutes to hours, and completely reversible. Dr. Fulwiler is a board-certified psychiatrist and will be present at all times during participant visits. He will be available to monitor for any psychological consequences

The primary risk to subjects of these procedures is that sensitive personal information would be mishandled resulting in a breach of confidentiality.

1. Potential Benefits to Subjects*

Participants may not benefit individually from participation in the study if the program does not help them with maintaining weight loss. Knowledge gained from this study may help others with similar conditions in the future.

1. Vulnerable Populations*

NA

1. Multi-Site Research*

NA

1. Community-Based Participatory Research*

NA

1. Sharing of Results with Subjects*

NA

1. Setting

We will recruit from the community by distributing flyers at the Weight Center at UMass Memorial Medical Center and weight loss programs and clinics throughout Central Massachusetts, and by placing advertisements on the internet (Craig’s list).

All procedures will be conducted at UMass Medical School.

Procedures:

MRI’s will be conducted in the **Advanced MRI Center** in the Radiology Department at UMMS. The Center also includes a nurses’ station, two patient holding rooms, two patient changing rooms with lockers, a staff changing room with lockers and a shower, and an animal surgery room with a holding room.

MBSR classes will be conducted in the UMMS **Center for Mindfulness in Medicine, Healthcare and Society** (CFM) directed by Dr. Saki F. Santorelli, the CFM is in the Division of Preventive and Behavioral Medicine, within the Department of Medicine at the University of Massachusetts Medical School.

HLC classes will be conducted in the the UMMS **Clinical Trials Unit**, a 2600 sq. ft. ambulatory site located on the 7th floor of the medical school in suite # S7-714.

Data Management & Analysis will be conducted in the **Center for Mental Health Services Research (CMHSR**) within the UMass Department of Psychiatry located on the 8th floor of Worcester State Hospital, the adjacent **Center for Comparative NeuroImaging (CCNI**), and the **Department of Quantitative Health Science**.

1. Resources Available

Personnel: The PI will devote 10% (1.2calendar months) in Year 1 and 20% (2.4 calendar months) in years 2 and 3 to the project. His research addresses the neuronal circuitry sub-serving emotion regulation and impulsivity. Over the last three years he has invested considerable time into a unique collaboration with the Mindfulness Center under the Direction of Dr Saki Santorelli and building a multidiscipline team of investigators to study Mindfulness-Based Stress Reduction (MBSR). Dr Fulwiler will be responsible for the design, implementation and presentation of the study. He will oversee all phases of recruitment, implementation of MBSR, data collection and outcome measurements, and will host monthly meetings with co-investigators to monitor progress on analyses and for setting agendas for the next steps. Dr. Fulwiler has extensive experience in neuroimaging studies of clinical populations.

The team of co-investigators includes several experienced clinical and health services researchers, experts in multi-modal MRI techniques including human fMRI imaging and resting state analysis, expertise in interventions for obesity and diabetes prevention and management, clinical epidemiology, biostatistics and statistical modeling for complex system analyses and behavioral therapy research with recruitment and tracking of large sample sizes. The co-I’s will each devote between 5-15% effort throughout the duration of the project.

A MBSR trainer will be hired who will devote 5% effort in year 1 and 8% effort in years 2 and 3. This will be an expert teacher from the Center for Mindfulness in Medicine at Umass Medical School and will be responsible for delivering the MBSR training

A research coordinator will be hired who will devote 20% effort in year 1 and 40% effort in years 2 and 3. This research coordinator (Masters level psychologist) will be added to the list of active study staff and will assist the investigators in subject recruitment, administering behavioral scales, data input and will be trained by its developer to provide the control intervention. Other responsibilities will include assisting in scheduling follow-up visits and general program support.

MRI Resources: The Advanced MRI Center in the Radiology Department at UMMS facilitates a new Philip’s 3.0T Achieva Quasar system which offers advanced shimming capabilities to obtain improved image quality. The Quasar Dual gradient system provides industry leading performance specifications for peak strength and slew rate with a dual mode capability that optimizes advanced applications requiring very high peak mode capabilities. The registered radiologic technologists with additional certification in magnetic resonance imaging, and the staff radiologists at the Central Massachusetts Magnetic Imaging Center (CMMIC) at UMMS are available for MRI research.

Computing resources: Computer hardware and software equipment are located in the CMHSR and CCNI. PCs are equipped with data management programs (e.g., Microsoft Access, Microsoft Excel, EndNote) for data coding and storage, statistical and qualitative data analysis software (e.g., SAS, SPSS, N6, STATA), and desktop publishing programs (e.g., Adobe PhotoShop, Quark Express, Microsoft Publisher). Network linkages for all staff include access to the internet and all university mainframes, resources of the Lamar Soutter Library and work stations. All offices and laboratories are networked with switched 10/100 Mb/s TCP/IP communication lines.

Data storage: Hard copy data are stored in locked file cabinets, in the locked offices of Principal Investigators (PI) in the CMHSR. Documents containing confidential information are filed separately from completed interview protocols. Electronic data files are stored on a UMMS intra-network server in folders with access limited to the PI and co-I’s. The data files are encrypted, and encryption keys are unique and changed on a regular basis, with access restricted to PIs and research staff.

All personnel have completed CITI training. All personnel involved in study enrollment, informed consent and data collection procedures complete formal training conducted by the PI prior to beginning study enrollment and will be instructed to call the PI on his cell phone should any questions arise during procedures.

1. Prior Approvals

NA

1. Recruitment Methods

Feasibility: An important goal of this study is to generate preliminary data for future large-scale randomized trials. Therefore, we will closely monitor study process using parameters suggested by Leon for clinical research.^[71]^ More specifically, we will track: (1) number of participants screened; (2) number of participants recruited; (3) number successfully randomized; (4) group-specific retention rates; (5) adherence to treatment protocols; and (6) intervention fidelity. Tracking these measures in real-time will allow us to make “mid-course” refinements in our study protocol as well as inform future study protocols. We will enroll 100 participants (50 randomized to each arm) and allow for up to 20% loss to follow up, for an effective sample size of 80. Our team has many years of experience doing clinical trials and behavioral therapy research with recruitment and tracking of large sample sizes, studying complementary and non-traditional approaches, including studies of wellness interventions, healthy eating/ obesity, stress/coping, and mindfulness. Recruitment will be conducted over a 2-year period using methods which have proven effective in our team’s previous clinical studies.

Advertisement: Flyers will be distributed and advertisements placed on the internet, including on www.craigslist.org and the UMMS Intranet. Also, we will advertise our study through our website (http://umassmed.edu/psychiatry/keepingoffweight.aspx) which was particularly created for the purposes of this study and we are also going to advertise this study in various social media. We are going to use exactly the same word context as in the advertisement flyer. Word of mouth will also be used. We will explain that we are conducting a study to compare two programs designed to help people who have lost weight keep it off and to study the effect these interventions on the brain with MRI.

Screening:

- 1. Telephone pre-screening: Individuals who respond to advertisements will be contacted by phone for a brief pre-screening which includes a brief description of the project and the exclusion criteria including MRI exclusion criteria (see Pre-Screen attachment). Subjects will be informed that their participation is voluntary and is for the purpose of research, and that if they do not appear to be eligible or are not interested in the study, their screening materials will be destroyed and only their names will be kept in a screening log. If they do appear to be eligible based on the pre-screen, they will be informed that the information collected will be kept in a locked file to maintain confidentiality until they meet with study personnel and will be kept separately from the contact page. The subject’s ID number will be added to the phone screen so the only way to link the information to the subject is via the ID number. Subjects who appear to be eligible will be sent the weight loss verification form by mail or email (their preference) and instructed to have their physician, weight loss counselor or fitness instructor sign it and bring with them to the first research visit.
  2. After the subjects have been prescreened by phone they will be scheduled for the first research visit. At this visit, the Research Assistant will confirm that the participant’s physician or weight loss counselor has signed the verification form, measure baseline height and weight and conduct the informed consent process. The study will be described in full detail, including the risks described above, and all of the subject’s questions will be answered. Subjects will have ample opportunity to reconsider their decision to participate. They will have the opportunity to opt out at the interview or at any point on the study day, including while the scan is underway. After the informed consent is signed a psychiatrist (P.I. or Co-I) will conduct a medical history and the Structured Clinical Interview for DSM-IV (SCID) to determine eligibility for the study. If subjects are not found to be eligible or are not interested in continuing with the study, they will be informed that the information collected during this visit will be destroyed. If for any reason, during the SCID interviews the participant is at imminent risk of harming him/herself or others, which can necessitate involuntary reporting and intervention, their potential loss for confidentiality will be discussed and disclosed to them at the time of consenting. If eligible, subjects will proceed with the baseline visit prior to randomization, including collection of demographic information, completion of psychological questionnaires, and will be escorted to the Advanced MRI Center where the MRI technician will repeat the MRI compatibility screen according to the Advanced MRI Center’s standard safety protocol. An independent neuroradiologist will review T1W 3D TFE sagittal and T1W FFE axial images for exclusion based on structural brain damage. Any medically important findings on the MRI will be identified and reported to the subject who will be given a referral, and at the participant’s request will also be reported to their physician. Any subject who is excluded based on the initial fMRI scan will be compensated for the initial visit and will be invited to continue with the intervention without data collection or additional compensation. For subjects who are found eligible, the Research Assistant will contact the person who signed the weight loss verification form for further verification.

No medical records will be used. All participants will be recruited from the community.

Compensation: Participants will receive $100 at the time of the data collection visits T0-T2. Subjects who complete only the informed consent and psychological measures at T0 or T1 will receive $25 (to cover time to complete questionnaires and travel time). Subjects who do not complete the study as a result of discomfort during scanning will still receive the full compensation.

1. Local Number of Subjects

100 subjects will be enrolled and all will be expected to complete the research procedures. We anticipate based on our previous experience with similar studies that approximately 200 subjects will need to be screened to identify 100 eligible subjects.

1. Confidentiality

Data will be stored by ID number; it will not include subject identifiers. Only the investigators will have access to identifiable data. The data for this study consist of MRI scans and questionnaires which will be coded with the subject’s identification number. Paper data will be stored in a locked file cabinet in the PI’s office, Rm 8B-1 at the Center for Mental Health Services Research, Bryan Building. The data will never be destroyed. The master list of subjects’ addresses and phone numbers will be destroyed at the end of the study. All study data will be stored without personal identifiers.

1. Provisions to Protect the Privacy Interests of Subjects (HIPAA)

No Protected Health Information will be collected.

All subjects will be reminded that they do not have to answer any questions they are uncomfortable answering and that they are free to end participation at any time.

1. Compensation for Research-Related Injury

This is a minimal risk from study participation and no funds have been set aside for coverage.

1. Economic Burden to Subjects

Subjects will be responsible for their transportation costs only. There are no known economic risks to subjects because of participation in the research.

1. Consent Process

We will follow SOP: Informed Consent Process for Research (HRP-090).

Eligible subjects will undergo the informed consent process conducted by the PI or Research Assistant in the Psychiatry Department or the Clinical Trials Unit (which are located across from each other on the 7th floor of UMass). The study will be described in full detail and all of the potential subject’s questions will be answered. Subjects will be informed about all of the risks described above and all aspects of the study. The consent process is expected to take 20-30 min. We will minimize the possibility of coercion or influence by providing subjects up to 1 hour to decide about participation and ample opportunity to reconsider their decision. They will have the opportunity to opt out at the initial phone contact, at the interview, or at any point on the study day, including while the study is underway. The PI or Co-I conducting the process will determine that the subject understands the information provided and is capable of making and communicating an informed consent. After the informed consent is signed subjects will be scheduled for the baseline visit.

**Non-English Speaking Subjects**

NA

**Waiver or Alteration of the Consent Process (consent will not be obtained, required information will not be disclosed, or the research involves deception)**

NA

**Subjects who are not yet adults (infants, children, teenagers)**

NA

**Cognitively Impaired Adults**

NA

**Adults Unable to Consent**

NA

**Adults Unable to Consent**

NA

1. Process to Document Consent in Writing

We will be following SOP: Written Documentation of Consent (HRP-091).

1. Drugs or Devices

NA

**References**

1. Kabat-Zinn, J., Full Catastropher Living: Using the Wisdom of Your Body and Mind to Face Stress. Pain and Illness, Delacorte, NY, 1990.

2. Santorelli, S.F., Heal Thy Self: Lessons on Mindfulness in Medicine. Random House/Bell Tower

1999.

3. Santorelli, S.F. and J. Kabat-Zinn, Mindfulness-Based Stress Reduction (MBSR) Professional Education and Training: MBSR Curriculum and Supporting Materials. 2011, Worcester, MA: Center for Mindfulness, Health Care and Society, University of Massachusetts Medical School.

4. Hajek, P., et al., Relapse prevention interventions for smoking cessation. Cochrane Database Syst Rev, 2009(1): p. CD003999.

5. Marcus, B.H., et al., Physical activity intervention studies: what we know and what we need to know: a scientific statement from the American Heart Association Council on Nutrition, Physical Activity, and Metabolism (Subcommittee on Physical Activity); Council on Cardiovascular Disease in the Young; and the Interdisciplinary Working Group on Quality of Care and Outcomes Research. Circulation, 2006. **114**(24): p. 2739-52.

6. Merrill, R.M., et al., Can newly acquired healthy behaviors persist? An analysis of health behavior decay. Prev Chronic Dis, 2008. **5**(1): p. A13.

7. Ory, M.G., et al., The science of sustaining health behavior change: the health maintenance consortium. Am J Health Behav, 2010. **34**(6): p. 647-59.

8. Cosci, F., et al., Nicotine dependence and psychological distress: outcomes and clinical implications in smoking cessation. Psychol Res Behav Manag, 2011. **4**: p. 119-28.

9. Elfhag, K. and S. Rossner, Who succeeds in maintaining weight loss? A conceptual review of factors associated with weight loss maintenance and weight regain. Obes Rev, 2005. **6**(1): p. 67-85.

10. Wing, R.R., et al., Maintaining large weight losses: the role of behavioral and psychological factors. J Consult Clin Psychol, 2008. **76**(6): p. 1015-21.

11. Bowen, S., et al., Mindfulness-based relapse prevention for substance use disorders: a pilot efficacy trial. Subst Abus, 2009. **30**(4): p. 295-305.

12. Chiesa, A. and A. Serretti, Mindfulness based cognitive therapy for psychiatric disorders: a systematic review and meta-analysis. Psychiatry Res, 2011. **187**(3): p. 441-53.

13. Hofmann, S.G., et al., The effect of mindfulness-based therapy on anxiety and depression: A meta-analytic review. J Consult Clin Psychol, 2010. **78**(2): p. 169-83.

14. Segal, Z.V., et al., Antidepressant monotherapy vs sequential pharmacotherapy and mindfulness-based cognitive therapy, or placebo, for relapse prophylaxis in recurrent depression. Arch Gen Psychiatry, 2010. **67**(12): p. 1256-64.

15. Hölzel, B.K., et al., How does mindfulness meditation work? Proposing mechanisms of action from a conceptual and neural perspective. Perspectives on Psychological Science, 2011. **6**: p. 537-559.

16. Liston, C., B.S. McEwen, and B.J. Casey, Psychosocial stress reversibly disrupts prefrontal processing and attentional control. Proc Natl Acad Sci U S A, 2009. **106**(3): p. 912-7.

17. Lewis, C.M., et al., Learning sculpts the spontaneous activity of the resting human brain. Proc Natl Acad Sci U S A, 2009. **106**(41): p. 17558-63.

18. Brewer, J.A., et al., Meditation experience is associated with differences in default mode network activity and connectivity. Proc Natl Acad Sci U S A, 2011. **108**(50): p. 20254-9.

19. Kilpatrick, L.A., et al., Impact of Mindfulness-Based Stress Reduction training on intrinsic brain connectivity. Neuroimage, 2011. **56**(1): p. 290-8.

20. Bjorntorp, P. and R. Rosmond, Obesity and cortisol. Nutrition, 2000. **16**(10): p. 924-36.

21. Lattimore, P. and L. Maxwell, Cognitive load, stress, and disinhibited eating. Eat Behav, 2004. **5**(4): p. 315-24.

22. O'Connor, D.B., et al., Effects of daily hassles and eating style on eating behavior. Health Psychol, 2008. **27**(1 Suppl): p. S20-31.

23. Wallis, D.J. and M.M. Hetherington, Emotions and eating. Self-reported and experimentally induced changes in food intake under stress. Appetite, 2009. **52**(2): p. 355-62.

24. Evers, K.E., et al., A randomized clinical trial of a population- and transtheoretical model-based stress-management intervention. Health Psychol, 2006. **25**(4): p. 521-9.

25. Mansyur, C.L., et al., Self-efficacy and barriers to multiple behavior change in low-income African Americans with hypertension. J Behav Med, 2012.

26. Ng, D.M. and R.W. Jeffery, Relationships between perceived stress and health behaviors in a sample of working adults. Health Psychol, 2003. **22**(6): p. 638-42.

27. Benninghoven, D., et al., Influence of anxiety on the course of heart disease after acute myocardial infarction - risk factor or protective function? Psychother Psychosom, 2006. **75**(1): p. 56-61.

28. Gariepy, G., D. Nitka, and N. Schmitz, The association between obesity and anxiety disorders in the population: a systematic review and meta-analysis. Int J Obes (Lond), 2010. **34**(3): p. 407-19.

29. Kuhl, E.A., et al., Relation of anxiety and adherence to risk-reducing recommendations following myocardial infarction. Am J Cardiol, 2009. **103**(12): p. 1629-34.

30. Castro, Y., et al., Structural and predictive equivalency of the Wisconsin Smoking Withdrawal Scale across three racial/ethnic groups. Nicotine Tob Res, 2011. **13**(7): p. 548-55.

31. Kahler, C.W., et al., Hostility in smokers with past major depressive disorder: relation to smoking patterns, reasons for quitting, and cessation outcomes. Nicotine Tob Res, 2004. **6**(5): p. 809-18.

32. Patterson, F., et al., Increase in anger symptoms after smoking cessation predicts relapse. Drug Alcohol Depend, 2008. **95**(1-2): p. 173-6.

33. Chiesa, A. and A. Serretti, Mindfulness-based stress reduction for stress management in healthy people: a review and meta-analysis. J Altern Complement Med, 2009. **15**(5): p. 593-600.

34. Grossman, P., et al., Mindfulness-based stress reduction and health benefits. A meta-analysis. J Psychosom Res, 2004. **57**(1): p. 35-43.

35. Biegel, G.M., et al., Mindfulness-based stress reduction for the treatment of adolescent psychiatric outpatients: A randomized clinical trial. J Consult Clin Psychol, 2009. **77**(5): p. 855-66.

36. Kabat-Zinn, J., et al., Effectiveness of a meditation-based stress reduction program in the treatment of anxiety disorders. Am J Psychiatry, 1992. **149**(7): p. 936-43.

37. Shapiro, S.L., K.W. Brown, and G.M. Biegel, Teaching Self-Care to Caregivers: Effects of Mindfulness-Based Stress Reduction on the Mental Health of Therapists in Training. Training and Education in Professional Psychology, 2007. **1**(2): p. 105–11.

38. Shapiro, S.L., G.E. Schwartz, and G. Bonner, Effects of mindfulness-based stress reduction on medical and premedical students. J Behav Med, 1998. **21**(6): p. 581-99.

39. Grossman, P., et al., MS quality of life, depression, and fatigue improve after mindfulness training: a randomized trial. Neurology, 2010. **75**(13): p. 1141-9.

40. Carlson, L.E. and S.N. Garland, Impact of mindfulness-based stress reduction (MBSR) on sleep, mood, stress and fatigue symptoms in cancer outpatients. Int J Behav Med, 2005. **12**(4): p. 278-85.

41. Gross, C.R., et al., Mindfulness-based stress reduction for solid organ transplant recipients: a randomized controlled trial. Altern Ther Health Med, 2010. **16**(5): p. 30-8.

42. Garland, S.N., et al., A non-randomized comparison of mindfulness-based stress reduction and healing arts programs for facilitating post-traumatic growth and spirituality in cancer outpatients. Support Care Cancer, 2007. **15**(8): p. 949-61.

43. Kieviet-Stijnen, A., et al., Mindfulness-based stress reduction training for oncology patients: patients' appraisal and changes in well-being. Patient Educ Couns, 2008. **72**(3): p. 436-42.

44. Jain, S., et al., A randomized controlled trial of mindfulness meditation versus relaxation training: effects on distress, positive states of mind, rumination, and distraction. Ann Behav Med, 2007. **33**(1): p. 11-21.

45. Fredrickson, B.L. and C. Branigan, Positive emotions broaden the scope of attention and thought-action repertoires. Cogn Emot, 2005. **19**(3): p. 313-332.

46. Tugade, M.M., B.L. Fredrickson, and L.F. Barrett, Psychological resilience and positive emotional granularity: examining the benefits of positive emotions on coping and health. J Pers, 2004. **72**(6): p. 1161-90.

47. Tugade, M.M. and B.L. Fredrickson, Resilient individuals use positive emotions to bounce back from negative emotional experiences. J Pers Soc Psychol, 2004. **86**(2): p. 320-33.

48. Wing, R.R., et al., A self-regulation program for maintenance of weight loss. New England Journal of Medicine, 2006. **355**(15): p. 1563-71.

49. O'Neil, P.M. and S. Rieder, Utility and validity of the eating behavior inventory in clinical obesity research: a review of the literature. Obes Rev, 2005. **6**(3): p. 209-16.

50. Paffenbarger, R.S., Jr., et al., Measurement of physical activity to assess health effects in free-living populations. Med Sci Sports Exerc, 1993. **25**(1): p. 60-70.

51. Buysse, D.J., et al., The Pittsburgh Sleep Quality Index: a new instrument for psychiatric practice and research. Psychiatry Res, 1989. **28**(2): p. 193-213.

52. Prochaska, J.J., B. Spring, and C.R. Nigg, Multiple health behavior change research: an introduction and overview. Prev Med, 2008. **46**(3): p. 181-8.

53. Van Dorsten, B. and E.M. Lindley, Cognitive and behavioral approaches in the treatment of obesity. Med Clin North Am, 2011. **95**(5): p. 971-88.

54. Cohen, S., T. Kamarck, and R. Mermelstein, A global measure of perceived stress. J Health Soc Behav, 1983. **24**(4): p. 385-96.

55. Radoff, L., The CES-D scale: A self-report depression scale for research in the general population. App. Psychol Meas, 1977(1): p. 385-401.

56. Spielberger, C.D., Manual for the State–Trait Anxiety Inventory (Form Y). 1983, Palo Alto, CA: Mind Garden.

57. Spielberger, C., State-Trait Anger Expression Inventory-2: Professional Manual. 1999: Psychological Assessment Resources, Inc.

58. Pavot, W., et al., Further validation of the Satisfaction with Life Scale: evidence for the cross-method convergence of well-being measures. J Pers Assess, 1991. **57**(1): p. 149-61.

59. Diener, E., et al., The Satisfaction With Life Scale. J Pers Assess, 1985. **49**(1): p. 71-5.

60. Gross, J.J. and O.P. John, Individual differences in two emotion regulation processes: implications for affect, relationships, and well-being. J Pers Soc Psychol, 2003. **85**(2): p. 348-62.

61. Niemeier, H.M., et al., Internal disinhibition predicts weight regain following weight loss and weight loss maintenance. Obesity (Silver Spring), 2007. **15**(10): p. 2485-94.

62. Grossman, P., Defining mindfulness by how poorly I think I pay attention during everyday awareness and other intractable problems for psychology's (re)invention of mindfulness: comment on Brown et al. (2011). Psychol Assess, 2011. **23**(4): p. 1034-40; discussion 1041-6.

63. Baer, R.A., et al., Using self-report assessment methods to explore facets of mindfulness. Assessment, 2006. **13**(1): p. 27-45.

64. Devilly, G.J. and T.D. Borkovec, Psychometric properties of the credibility/expectancy questionnaire. J Behav Ther Exp Psychiatry, 2000. **31**(2): p. 73-86.

65. Frison, L. and S.J. Pocock, Repeated measures in clinical trials: Analysis using mean summary statistics and its implications for design. Statistics in Medicine, 1992. **11**: p. 1685–1704.

66. Fritz, M.S. and D.P. MacKinnon, Required Sample Size to Detect the Mediated Effect. Psychological Science, 2007. **18**(3): p. 233-239.

67. Groenwold, R.H., et al., Dealing with missing outcome data in randomized trials and observational studies. Am J Epidemiol, 2012. **175**(3): p. 210-7.

68. Little, R. and D. Rubin, Statistical Analysis with Missing Data, Second Edition. 2002, New York: John Wiley and Sons.

69. Schafer, J.L., Multiple imputation: a primer. Stat Methods Med Res, 1999. **8**(1): p. 3-15.

70. Long, Q., C.H. Hsu, and Y. Li, Doubly Robust Nonparametric Multiple Imputation for Ignorable Missing Data. Stat Sin, 2012. **22**: p. 149-172.

71. Leon, A.C., L.L. Davis, and H.C. Kraemer, The role and interpretation of pilot studies in clinical research. J Psychiatr Res, 2010.
